# Extracellular Vesicles as Natural, Safe and Efficient Drug Delivery Systems

**DOI:** 10.3390/pharmaceutics11110557

**Published:** 2019-10-28

**Authors:** Federico Villa, Rodolfo Quarto, Roberta Tasso

**Affiliations:** 1 U.O. Cellular Oncology, Ospedale Policlinico San Martino, 16132 Genova, Italy; vilfed@libero.it (F.V.); rodolfo.quarto@unige.it (R.Q.); 2 Department of Experimental Medicine, University of Genova, 16132 Genova, Italy

**Keywords:** extracellular vesicles, isolation methods, drug carriers

## Abstract

Extracellular vesicles (EVs) are particles naturally released from cells, delimited by a lipid bilayer, carrying functionally active biological molecules. In addition to their physiological role in cellular communication, the interest of the scientific community has recently turned to the use of EVs as vehicles for delivering therapeutic molecules. Several attempts are being made to ameliorate drug encapsulation and targeting, but these efforts are thwarted if the starting material does not meet stringent quality criteria. Here, we take a step back to the sources and isolation procedures that could guarantee significant improvements in the purification of EVs to be used as drug carriers, highlighting the advantages and shortcomings of each approach.

## 1. Introduction

Extracellular vesicles (EVs) are cell-derived membrane vesicles that represent an endogenous mechanism for intercellular communication [1]. EVs can be classified in exosomes, nano-sized vesicles (with a diameter in the range of 30 to 120 nm), that originate from the cell endocytic compartment through the formation of multivesicular bodies (MVB) [2], microvesicles, with a diameter up to 1 μm released by cell membrane budding, and apoptotic bodies, with a dimension similar to platelets, derived from blebbing of dying cells. Despite the distinct sizes and biogenesis, the absence of standardized isolation methods and the numerous similarities existing between these two subclasses make it particularly challenging to distinguish among them. In this review, we will use the generic term EVs to indicate both exosomes and microvesicles.

EVs have been shown to carry functionally active biological materials including proteins, mRNAs and miRNAs, which makes them capable of transmitting signals to target cells in the surrounding environment as well as to distant organs, via blood and lymphatic vessels [3].

The scientific community has recently turned its interest toward the evaluation of EVs as drug delivery vehicles [4]. In this context, EVs offer significant advantages over current drug delivery systems, such as liposomes and polymeric nanoparticles [5]. Since EVs can be obtained with an autologous procedure from the patient’s cells or blood, they do not solicit the immune system as usual synthetic formulations do. In addition, given their phospholipid bilayer, EVs can directly fuse with the targeted plasma membrane, thus allowing a more efficient internalization of the encapsulated drug [6,7]. Their hydrophilic shell, together with the presence of anti-phagocytosis surface markers (i.e., CD47), enables them to evade phagocytosis by monocytes and macrophages of the reticulo-endothelial system, hence weakening their clearance [8,9]. Moreover, the limited size allows them to efficiently extravasate through the inter-endothelial junctions and fenestrations of both existing and neo-synthesized vessels [10]. Thanks to these peculiar characteristics, they can spread and accumulate in the parenchyma of solid tumors [11]. EV biological properties derive from their sophisticated membrane structure, characterized by the presence of several proteins for active targeting, adhesion, cell fusion, and intracellular release of their content [12]; this functional complexity is not easily reproduced by de novo synthesized nanoparticles. Among the several types of nano-based drug delivery systems, liposomes are probably the most used, due to their non-toxicity and capacity to accommodate high amounts of compounds. However, a still inadequate in vivo targeting efficiency together with a potential immunogenicity associated to liposomal formulations has limited their broad applicability in therapeutics [13].

Therefore, the greater specificity combined with the consequent limited induction of systemic side effects make EVs ideal vehicles for drug delivery [13]. Although this research field is in its infancy, in the last decade, the use of bio-engineered EVs for the delivery of cytotoxic molecules in preclinical models has produced encouraging results [14,15,16,17,18]. These experimental evidences, in addition to preliminary clinical data, indicate that EV formulations may not only enhance the safety and biodistribution of commonly used drugs, but also increase their efficacy. For example, it has been demonstrated that the administration of EV-encapsulating chemotherapy drugs leads to a significant reduction of drug accumulation in off-target organs, thus preventing important side effects during standard clinical protocols [14]. An astonishing number of newly published papers perfectly depict the scientific community’s authentic interest in this flourishing research field.

Nevertheless, the first aim of this review is to disclose an overview of the latest applications of EVs as drug delivery vehicles, focusing on the sources employed, the molecules selected, and the final intended targets. In the second part of the review, we will take a step back and bring attention to the basics of one of the main pitfalls concerning EVs: the urgent need for highly pure vesicle preparation.

## 2. *EVs* as Drug Delivery Vehicles: State of the Art

### 2.1. Sources of EVs 

Most literature data dealing with EVs as drug carriers have been published employing vesicles released by in vitro cultured cells. Since the expression of specific cell surface markers, that has been shown to influence the EV biological activity and the subsequent therapeutic effect is strictly related to the parental cell, different cell sources have been investigated. These include model cell lines, such as HeLa and HEK 293, various tumor cell lines, primary cultures of dendritic cells, and mesenchymal stromal cells.

The model cell lines have been selected as an EV source in those cases in which specific EV targeting was obtained through the transfection of the donor cells or a huge amount of EV was requested. This approach has been adopted, for example, to deliver HEK 293-derived EVs loaded with either miRNAs [19] or chemotherapeutic drugs [20], demonstrating, in these cases, a more efficient cellular uptake and biodistribution in comparison to both free or liposomal formulations of the same drugs.

The rationale underlying the use of tumor-derived EVs as advanced drug delivery systems is to exploit their tumor-specific integrin expression pattern that could guarantee an efficient organotropism [21]. It has been recently demonstrated by Garofalo and colleagues that EVs derived from the A549 cancer cell line can deliver an encapsulated oncolytic virus with a specific tropism to tumors induced in mice by the injection of the same EV donor cell line [22]. An interesting paper published in 2016 reports that EVs derived from the EL-4 mouse lymphoma cell line were mixed with a potent anti-inflammatory compound to enhance its efficacy. The authors demonstrated, both in vitro and in vivo, that the incorporated molecule possessed increased solubility, stability, and bioavailability [23]. Despite these encouraging results, it is important to underline that all co-purifying components in enriched vesicle fractions from tumor sources could be potentially transferred to target cells. For this reason, alternative and safer sources should be taken into consideration. In particular, the selected cell should guarantee an efficient production of non-immunogenic EVs to prevent potential adverse effects after administration. Immature dendritic cells (DCs) could represent an ideal source, as they have been reported to be immunologically inert [24]. It has been reported that DC-derived EVs loaded with siRNA were targeted to the mouse brain, specifically delivering their content to neurons [25]. The same cell source was used to produce EVs incapsulated with chemotherapeutics to be delivered to tumor tissues in vivo [16].

Another explored source of EVs is represented by mesenchymal stromal cells (MSCs). MSCs are a heterogeneous cell population present in many tissues able to differentiate into mesodermal lineages and endowed with an immunomodulatory potential. Recent studies have demonstrated that MSCs exert their immunosuppressive function, secreting EVs that can deliver their cargo to target cells without inducing oncogenic or immunogenic effects [26]. EVs isolated from MSCs have been loaded with anti-neoplastic drugs, demonstrating an increased cytotoxic effect and target specificity [27,28,29]. Many efforts have been made to load miRNAs into MSC-derived EVs that could represent effective strategies for the treatment of different tumor types [30,31,32,33].

More recently, a paper by Sancho-Albero and colleagues reported the intriguing possibility of loading hollow gold nanoparticles in EVs secreted by human placental MSCs to be selectively targeted to specific cell types by light-induced hyperthermia [34].

### 2.2. EV Loading Methods

EV physiological properties together with their immune “stealth” characteristics have been extensively exploited to safely deliver molecules to specific target cells bypassing complex biological barriers and even enhancing their therapeutic effects. The EV loading process of specific cargos can be achieved by manipulating already isolated EVs (exogenous loading) or acting on parental cells (endogenous loading).

#### 2.2.1. Exogenous Loading

The exogenous EV loading methods are recommended when a biological modification of the parental cells is not feasible. Indeed, such methods are more customizable and may broaden the horizons of EV applications to a wider variety of EV sources.

Various approaches have been described to exogenously incorporate therapeutic agents into isolated EVs, ranging from the simple vesicle incubation with both lipophilic molecules and hydrophobically modified compounds, to the application of active loading techniques, such as repeated freezing-thawing procedures and permeabilization with saponin, extrusion, sonication, and electroporation [35].

In the last years, by implementing and refining the aforementioned methods, it has been possible to test the therapeutic effect of a wide palette of vesicle-internalized components, encompassing small molecules, chemotherapeutic drugs, siRNAs, miRNAs, DNA, and proteins.

Pre-clinical studies have demonstrated that the chemotherapeutic drugs doxorubicin [16,20] and paclitaxel [36,37] encapsulated in EVs presented an improved biodistribution and efficacy in both in vitro and in vivo assays. These enhanced effects are due to a more specific and direct accumulation of the cytotoxic molecules in tumor cells together with a reduction of side effects in off-target organs. Based on these promising results, it could be envisaged that a number of molecules with a potential great therapeutic effect, but characterized by limited bioavailability, poor absorption, quick metabolism, and rapid systemic elimination, could benefit from being encapsulated in EVs [23].

Moreover, it has been reported that the delivery of anti-inflammatory agents could represent a promising non-invasive approach for the treatment of brain inflammatory-related diseases such as glioblastoma and experimental autoimmune encephalomyelitis. This assumption is based on recent data indicating how intranasally administered EVs can effectively deliver curcumin to the brain without observable side effects, thus being able to penetrate the blood–brain barrier [38].

EVs can be loaded with miRNAs or siRNAs and are able to effectively deliver the genetic material to cancer cells. Mendt and colleagues generated clinical-grade EVs employing good manufacturing practice (GMP) standards. EVs have been derived from bone marrow-MSCs and loaded by electroporation with a siRNA-targeting oncogenic Kras *(iExosomes).* This EV preparation is able to suppress cancer growth and increase the survival of mice bearing pancreatic ductal adenocarcinomas [39]. Recently, the application of *iExosomes* resulted in a new clinical trial for the treatment of metastatic pancreatic cancer (NCT03608631). This promising result, together with the work of other laboratories, provide evidence for the concept of using EVs as natural, safe and efficient drug delivery vehicles for RNA-based therapeutics in anti-cancer applications [40].

EVs have proven to be extremely efficient in delivering their cargo inside aggressive cancer cells that rely on the process of macropinocytosis to sustain their fast growth rate, getting a proper influx of nutrients. Nakase and colleagues tried to exploit this non-selective cellular getaway to deliver EV-encapsulated proteins directly in the cytoplasm of cancer cells. Indeed, they have shown how the EV encapsulation of the ribosome-inactivating protein saporin together with epidermal growth factor (EGF) can induce a specific and increased cytotoxicity, resulting in the growth inhibition of cancer cells [41].

A recently developed approach explored the feasibility of suppressing protein expression in cancer cells via Clustered Regularly Interspaced Short Palindromic Repeats (CRISPR)-associated endonuclease (Cas)9-mediated gene editing. Till now, the most used systems to introduce the plasmid for the expression of Cas9 and the chimeric single guide RNA (sgRNA) in cancer cells were represented by viral vectors [42]. Despite their high loading capacity, this type of virus could trigger immunogenic reactions and display toxicity. Kim and colleagues tested a new in-vivo delivery vehicle for the disruption of Poly (ADP-ribose) polymerase-1 (PARP-1) expression by using tumor-derived EVs loaded with Cas9 and PARP-1 sgRNA-encoding plasmids. This innovative EV-based approach has proved to be effective, being able to inhibit ovarian cancer proliferation by activating an apoptotic pathway in SKOV3-derived tumors in mice [43].

However, it is important to underline that the definition of a gold standard technique for the EV cargo incorporation has not been reached yet. The above-mentioned examples of exogenous loading methods, being strictly dependent on the characteristics of both cargo and EV source, have led to contrasting results and variable loading efficiencies [44].

#### 2.2.2. Endogenous Loading

The endogenous loading techniques exploit the natural cellular machinery to sort and load the desired molecules or genetic material into EVs during their biogenesis.

The chemotherapeutic drugs paclitaxel and doxorubicin were easily incorporated, incubating the parental cells with moderate doses of exogenous molecules [27,45]. The EV-encapsulated drugs were then isolated from the cell conditioned media after appropriate exposure and washing steps. The feasibility of this procedure has been successfully demonstrated by different research groups testing the incorporation of various molecules. However, the simple and linear rationale behind this idea could be spoiled by a low drug loading efficiency together with further complications due to drug-induced cytotoxicity on donor cells.

An alternative method to endogenously load therapeutic miRNAs into EVs has been described. The EV-producing cells can be transfected with a plasmid encoding the desired miRNA precursor in order to induce its overexpression and subsequent encapsulation in EVs. The miRNA-enriched EVs have been successfully used by several laboratories to transfer their genetic cargo to target cells both in vitro and in vivo [19,30,33]. Furthermore, Yuan and colleagues have tried to combine the remarkable and selective anticancer properties of the tumour necrosis factor-related apoptosis inducing ligand (TRAIL) to the therapeutic potential of EVs. The soluble recombinant form of TRAIL (rTRAIL) has been already extensively tested as a cancer therapeutic agent but its therapeutic benefit has been spoiled by a poor pharmacokinetic. In order to overcome this hurdle, the authors have established TRAIL-transduced MSCs (MSCTRAIL cells) with lentiviruses expressing human TRAIL. EVs secreted by MSCTRAIL cells express on their membrane TRAIL molecules and have been demonstrated to induce apoptosis in cancer cells with high efficiency and selectivity [46].

Another field that could greatly benefit from the use of EV-mediated delivery is the oncolytic virotherapy against cancer. Although these viruses are engineered to efficiently infect and kill cancer cells, it has been reported that viral particles can be detected by host immune system and inactivated by neutralizing antibodies, hampering viral replication and decreasing the treatment efficacy. Garofalo and colleagues, in the wake of the Trojan exosome hypothesis [47], have recently investigated the possibility of encapsulating oncolytic adenoviruses into EVs in order to utilize them as immuno-stealth carriers for targeted viral delivery to cancer cells [22]. After in vitro infection of A549 cells with the oncolytic adenovirus Ad5D24CpG, they isolated the EV-encapsulated virus (EV-Virus) naturally secreted in the conditioned media, inactivating any free, not EV-encapsulated, virus present in the preparation by an NaOH treatment. They have further shown how the systemic delivery of oncolytic virus with paclitaxel, both encapsulated in EVs, resulted in improved drug efficacy and reduced off-target toxicity in nude mice bearing A549-derived subcutaneous tumors [48].

### 2.3. EV Targeting

In order to significantly improve both the delivery and the biodistribution of their content, EVs can be engineered by anchoring peptides on their membrane that recognize specific cell surface receptors [19,25,49].

The “surface display” has been one of the first technologies to generate targeted EVs and requires genetic modification of the secreting cells [50]. Cells can be engineered by transfection/transduction with a vector encoding for a targeting peptide to be displayed on the surface of the vesicular membrane as a chimeric protein with an EV-sorting domain represented by the transmembrane and intravesicular domain of an ubiquitous EV membrane protein (i.e., Lamp2B, lactadherin). Such engineered construct will allow vesicle-specific targeting [51,52,53].

The functionalization of EV surface can be further performed after their isolation. Smyth and colleagues successfully used the click chemistry to conjugate Azide-fluor 545 to EVs chemically modified with alkyne groups. Thanks to its high efficiency and the mild reaction conditions requested, the authors proposed this methodology as a simple tool to label EVs with fluorescent, radioactive, and MRI agents for their in vivo tracking [54].

In another study, Antes and colleagues described an EV membrane engineering methodology, termed “cloaking”, to directly embed EV surfaces ex vivo with a modified glycerol-phospholipid-PEG (DMPE-PEG) anchor conjugated to streptavidin. DMPE-PEG can be used as coupling point for biotinylated molecules, such as fluorescent molecules, targeted antibodies and tissue-homing peptides, enhancing EV-specific uptake and biodistribution [55].

## 3. Plasma-Derived EVs

The efforts made to load bioactive molecules into EVs and engineer their membrane could be in vain or lead to unsatisfactory outcomes if the starting EV population does not fulfill some fundamental characteristics of quality and purity [56]. This aspect is strictly related to the source selected for EV isolation. Many studies dealing with the use of EVs as drug carriers have been published employing vesicles released by in vitro cultured cells [5,13]. The known chemical composition of the culture media and the possibility of using serum-free conditions might result in an easier isolation of EV populations [57]. However, the difficult logistics and the costs for the ex vivo cell expansion in highly qualified structures is not affordable by both public and insurance-driven health systems. Moreover, the translation of a research-grade cell culturing process into a scalable, clinical-grade protocol is expensive, time-consuming, and vulnerable to contamination [58]. Therefore, the use of biological fluids, such as plasma or serum, as alternative sources, offers undeniable advantages [56]: (i) they are particularly enriched with EVs, reaching 1–5 × 10^8^ vesicles/mL, even if the yield depends on the isolation protocol adopted [59]; (ii) plasma and serum are “ready to use” and “cheap” sources, allowing to obtain a huge amount of vesicles without the need of setting up in vitro cell cultures; (iii) EV isolation can be realized with an autologous procedure (the patient’s own blood can be used), a more advisable choice to prevent immune-mediated adverse reactions. In the case of patients debilitated by severe diseases or under chemotherapy regimens, fresh frozen plasma (FFP) from compatible healthy donors could be used as a clinical translational source for vesicle isolation.

However, even these sources present some drawbacks that make EV isolation trickier and technically challenging. This is mainly due to the presence of abundant protein mixtures with a wide range of concentration, protein-nucleic acid aggregates, and subcellular fragments, that, all together, could literally coat the EV membranes causing their aggregation during the isolation process, thus influencing not only the recovered yield, but, more importantly, altering their biological activity [60,61]. Further complicating this scenario are blood lipoproteins that possess similar size and densities to small vesicles and are so abundant in both plasma and serum (~10^16^/mL) [62,63]; they could contaminate the preparation of EVs in a way that may even invalidate the reliability of downstream functional experiments dealing with EV tracking analysis [64]. Indeed, the most used approaches to evaluate vesicle uptake/internalization by target cells involves the labeling of vesicles with fluorescent lipophilic dyes (PKH26 or PKH67 [65,66], DiD and DiR [39,67], CellMask [68], etc.); these molecules can be incorporated into any lipid structure, and none of them is specific for the vesicular membrane [69]. Both protein and lipoprotein contaminants can significantly contribute to lipophilic dye retention and transfer to acceptor cells [64], potentially generating undesired experimental artifacts.

It is becoming increasingly clear that the procedure employed to isolate plasma/serum-EVs is really challenging and the desired quality and purity of the vesicle preparation is unlikely to be achieved if the standard procedures developed to retrieve vesicles from conditioned media are adopted [70].

In any case, regardless of the selected isolation method, the pre-processing steps are crucial when considering serum or plasma samples. It is in fact recommended to use fresh plasma whenever possible [71]. If fresh frozen plasma is the only possible option, an essential expedient is to perform extra-centrifugations at low-speed followed by ultrafiltration (0.22 μm) before freezing and after thawing the sample. Indeed, if plasma is frozen immediately after low-speed centrifugation, larger floating vesicles will be damaged by the freezing process, releasing their content of nucleic acids and proteins that combine in string-like aggregates, potentially spoiling the yield and quality of the whole process [56].

In the last years, many research groups have attempted to define a proper gold standard for the purification of plasma/serum-EVs to be used as drug carriers and, consequently, a flourishing number of protocols and techniques have been recorded in the scientific literature. Herein, we will focus on the latest and most promising results attesting to this scientific effort.

## 4. EV Isolation Techniques

A consensus agreement regarding the gold-standard method for the isolation and purification of biofluid-derived EVs still lacks and is strictly dependent on the downstream applications. A multitude of commercially-available methods exist [72,73,74]. These kits guarantee a simple and fast EV isolation procedure, but most of them are only applicable to high-throughput biomarker-type studies (especially miRNA signature). Indeed, if plasma/serum-derived EVs are intended to be modified for therapeutic purposes, they have to fulfill some essential biological characteristics: the vesicle preparation has to be pure; morphologically intact; functionally active; and free of plasma proteins, lipoproteins and nucleic acids [56].

Since the dawn of their discovery, EV isolation has mainly ruled out adopting protocols inherited from other scientific disciplines, such as virology and biochemistry. These include ultracentrifugation (UC), density gradient centrifugation, size exclusion chromatography (SEC), and ultrafiltration (although the latter is not fully disclosed to the general scientific community due to deformation and breaking up of vesicles which may potentially skew the results of downstream analysis) [70].

Such techniques are still widely used by most laboratories worldwide. However, without the appropriate refinements, they can be suitable methods only for isolating EVs to be employed as they are, without any further handling. If vesicles have to be used as drug carriers, after isolation they are extensively manipulated to encapsulate the drug/bioactive molecule, and, in this case, particular attention has to be paid to the quality of the starting isolated material.

Newer techniques have been developed, such as acoustic sorting and nanowired-on-microcapillary trapping [75,76]; however, their robustness, reliability, and compatibility with EV purification are not yet fully embraced by the scientific community. Moreover, the required devices and expertise may be unaffordable for many research groups.

Below, we will report a summary of the progresses made for the isolation of biofluid-derived EVs to be used as drug vehicles, focusing on the pros and cons of each technique (Table 1).

### 4.1. Ultracentrifugation

Differential ultracentrifugation (UC) is undoubtedly the most applied method for isolating EVs and has been considered for a long time as the gold standard [77,78]. This technique involves a variable number of centrifugations at increasingly higher speeds and longer times in order to pellet sequentially smaller particles, till reaching EVs in the last steps at 100.000/120.000× *g*. UC is a versatile tool that allows the modification of some parameters, such as speed, temperature or rotor type in order to meet the requirements associated with the different downstream applications [79].

However, it is of capital importance to be aware of the main pitfalls that are hidden even in an apparently simple procedure like UC, especially when we consider vesicles derived from biofluids. As stated above, plasma or serum have a different chemical and molecular composition compared to culture media, and the high protein concentration could significantly affect the sedimentation efficacy [80].

Indeed, if a standard UC protocol is applied to a highly viscous biofluid such as plasma, the efficiency of EV isolation is less than 5% [81]. It is possible to overcome this unsatisfactory yield by applying some expedients, i.e., diluting the plasma to reduce the viscosity of the solution, or increasing the speed and duration of UC (3–14 h) [82]. Nevertheless, it has been reported by several studies that repeated ultracentrifugation steps at higher speed may not only damage the vesicles, changing their morphology and reducing their biological activity, but even cause a massive vesicle aggregation in clusters that are highly heterogeneous in size and number [83]. Albeit present, these aggregates could not be detected by the standard methods used for characterizing the purified EVs [84,85], such as nanoparticle tracking analysis (NTA) [86,87] or tunable resistive pulse sensing (tRPS) [88]: in fact, the micrometer-large aggregates of vesicles could be out of the instrument range for an optimal tracking or their strong scattering intensity could be considered as noise and, therefore, not included in the analysis. However, it has been recently reported that the addition of the non-reducing disaccharide sugar trehalose into both isolation and storage buffers, helps to maintain the dispersal of EVs during the UC process, reducing the formation of aggregates and preserving their integrity and stability during the storage and the following freezing-thawing cycles [89]. In any case, all the innovative EV preservation strategies have been recently reviewed by Kusuma and colleagues [90].

Another important aspect that has to be considered to preserve physical characteristics of EVs is the correct choice of rotor type and its proper usage [79]. The most commonly used rotors are the swinging bucket (SW) and the fixed angle (FA) rotors; they profoundly differ in terms of performance, k-factor (clearance factor) and pelleting efficiency. For example, SW rotors possess longer sedimentation path length than FA ones. Although this characteristic is responsible for the decreased pelleting efficiency, SW rotors are considered the best choice by the scientific community, being the most suitable support for the separation of particles with similar sedimentation coefficients [79,91].

### 4.2. Density Gradient UC

A strong improvement in terms of EV quality and purity has been observed by applying a iodixanol/sucrose density gradient or a sucrose density cushion centrifugation protocol [92,93]. EV, and in particular, exosome density (1.15 to 1.19 g/mL) is similar to sucrose or iodixanol, whose density produces a cushioning effect, maintaining the integrity of EVs and separating protein contaminants of high density (1.22 g/mL) [94].

The density gradient is generated, overlaying increasing concentrations of sucrose or iodixanol in a centrifuge tube. Plasma or serum samples are then placed on top of the gradient and centrifuged at higher forces (greater than 150,000× *g*) compared to standard UC. Vesicles sneak through the gradient until reaching the point at which their density matches with the one of the surrounding sucrose/iodixanol, outrunning the contaminating protein along their run [95].

The sucrose cushion centrifugation is a similar technique where vesicles are pelleted on a more dense sucrose solution; in this way, it is possible to gently concentrate the sample, since the mechanical stress is reduced and the resulting particles remain morphologically intact [94].

However, these techniques are laborious, time-consuming, conceal some technical difficulties and, more importantly, only small volumes of biofluids can be processed. Moreover, it has recently been argued that density gradient centrifugation is not able to completely separate vesicles from APOB^+^ material (i.e., APOB^+^ lipoproteins and blood HDLs, that own similar densities) [62,96].

### 4.3. Size Exclusion Chromatography (SEC)

Recently, size exclusion chromatography (SEC) has become an increasingly popular technique to purify EVs from biofluids, and in particular, plasma [70,97,98].

SEC is a simple and fast procedure characterized by a low infrastructural demand, it is customizable since different types of matrices/resins can be used (e.g., Sepharose 2B, CL-4B, Sephacryl S-400), good quality columns are commercially available, and it is possible to scale the process up to more than 100 mL of plasma processed in one step [97,99,100].

A small inherent disadvantage of SEC is that it does not concentrate samples (the fractions obtained are of the order of magnitude of the milliliter) and therefore, if the downstream procedures require EVs in small volumes, a second step is necessary [101,102]. Moreover, the co-purification of aggregates of the same size could hamper the real efficiency of the SEC-based method and could be the cause of contrasting results obtained by different laboratories (in terms of yield and protein contamination grades). As described by Hong and colleagues, a proper optimization of SEC-based protocols is therefore needed, paying particular attention to crucial steps (column setup, loaded sample volume, fraction collection) that could be the cause of the variability associated with the method [103].

## 5. Combined Protocols: Are they the Best Solution?

As mentioned above, an exhaustive purification of plasma/serum-derived EVs from contaminating proteins and lipoprotein particles is an arduous exercise. UC, density gradient UC and SEC are certainly good isolation techniques, but, as discussed above, present serious shortcomings, which, in some cases, are not compatible with the levels of yield, purity and quality required when EVs are intended to be used as drug carriers for therapeutic purposes.

The use of multiple and combined isolation techniques could represent a significant step forward [104,105]. Assembling together the unique and valuable characteristics of each method could be the key to obtain purer vesicle preparations. Although the requirement of long procedures involving multiple steps is expected to adversely affect the vesicle yield [96], the potential loss of material could be rewarded by a substantial benefit in terms of quality and purity.

Some laboratories are playing a pioneering role in this evolving field, and different combined isolation approaches have been recently tested. Corso and colleagues tested a novel core bead liquid chromatography technique for EV purification that combines size separation with bind-elute chromatography (BE-SEC) [106]. Applying this approach, the purification of high-quality vesicles from large volumes of conditioned media can be obtained. BE-SEC is fast, reliable and scalable, and, more importantly, provides isolation of non-fused, intact vesicles. 

The use of a two-step isolation procedure, combining density cushion separation followed by SEC, has been proposed [105]. This approach was elegantly demonstrated to be useful for the isolation of pure EVs from proteinaceous contaminants, as demonstrated by subsequent electron microscopy and mass spectrometry analysis. Indeed, 1187 specific proteins were identified, without undesirable contamination of plasma proteins and lipoprotein particles.

Moreover, with the common aim of processing large volumes of plasma/serum-EVs, free of non-relevant proteins and without running the risk of losing precious material, it has been also proposed to introduce some essential modifications to a conventional and dated purification method [107]. After cleaning the plasma with a low-speed centrifugation and filtration step (0.22 μm), the authors isolated EVs on a Sepharose 2B size-exclusion chromatography column; the collected fractions were subsequently pelleted by UC. After checking by Western blot EV profiles, the authors concluded that this method leads to the recovery of morphologically intact vesicles, largely depleted of contaminating immunoglobulins and still able to mediate intercellular communication [56]. Further modifications associated with this combined method have been proposed. Instead of pelleting EVs by UC, Lobb and colleagues recommended the use of specific protein concentration devices (centrifugal filters with a regenerated cellulose membrane with a pore size of 10 kDa) to rapidly and gently concentrate vesicle fractions derived from SEC in an efficient time frame [101]. Another further improvement in EV purification has been tested combining UC and affinity chromatography on Sepharose-bearing immobilized antibodies against vesicle surface proteins, such as CD9, CD63, and CD81 [108,109]. This additional purification step leads to the obtaining of significantly purer preparations, as demonstrated by the absence of appreciable impurities. Although these results were obtained using placenta as an EV source, it could be envisaged to apply similar techniques starting from plasma/serum samples.

An alternative method for EV purification has been described, which utilizes ultrafiltration followed by vesicle capture on heparin-affinity beads [110]. This, like others magnetic separation strategies, provides satisfactory results mainly for RNA extraction. Some downstream applications require a more precise purification of the starting material. 

The attempt to use polymer-based EV-precipitation kits (Exo-spin) before SEC separation should also be mentioned [81]. However, the scientific community is somewhat skeptical about the usage of precipitation techniques due to the very low purity achieved [96,111].

It is of capital importance to highlight that, regardless of the method or the combination of techniques used to isolate EVs for therapeutic purposes, the purity grade of the final vesicle preparation has to be carefully proved and quantified at both the molecular and colloidal length scales, by relying on different approaches, ranging from classical bioanalytical methods (i.e., Bradford, Western Blot, immunoassays for APOB^+^ lipoproteins) to more sophisticated biophysical techniques (NTA, tRPS, scanning Helium Ion Microscopy, and flow cytometric analysis) [96,112,113].

Taken together, these new studies indicate how imperative the use of appropriate combined approaches is, exploiting the advantageous characteristics of each procedure, when working with plasma and other biological fluids. Indeed, the employment of these composite sources introduces a new level of complexity to EV isolation, and this has to be particularly deemed when the end-point application is the use of vesicles as drug carriers and delivery vehicles. Isolates produced with these purposes should in fact undergo other tricky downstream manipulations (i.e., electroporation, sonication, direct transfection) that could potentially interfere, by themselves, with the final outcome.

## Figures and Tables

**Table 1:** Advantages and shortcomings associated with the most-commonly used isolation methods of biofluid-derived EVs.

ISOLATION METHOD	PROS	CONS	EV YIELD
*Ultracentrifugation (UC)*	versatile cost-effective vesicle enrichment as pellet	time-consuming low purity aggregation with “contaminating” proteins	Medium (prolonged ultracentrigugations needed, with consequent aggregate formation)
*Density gradient UC*	vesicle subtypes isolation vesicle enrichment as pellet	time-consuming laborious small sample volumes low purity	Low (the amount of starting material is limited; possible EV loss during fractionation)
*Size exclusion chromatography (SEC)*	reproducible preservation of integrity and activity	specific equipments long run times low sample volume	Medium (part of EVs can elute with contaminating proteins)
*Ultrafiltration*	cost-effective	time-consuming low accuracy deformation and breaking up of vesicles	Low (not applicable directly to biofluids)
*Commercial kits*	rapid easy to use do not require special equipments	high costs for large sample volumes low accuracy	High (co-isolation of contaminants)
